# One-Year stable perovskite solar cells by 2D/3D interface engineering

**DOI:** 10.1038/ncomms15684

**Published:** 2017-06-01

**Authors:** G. Grancini, C. Roldán-Carmona, I. Zimmermann, E. Mosconi, X. Lee, D. Martineau, S. Narbey, F. Oswald, F. De Angelis, M. Graetzel, Mohammad Khaja Nazeeruddin

**Affiliations:** 1 Group for Molecular Engineering of Functional Materials, Institute of Chemical Sciences and Engineering, Ecole Polytechnique Fédérale de Lausanne, Sion CH-1951, Switzerland; 2 Computational Laboratory for Hybrid/Organic Photovoltaics (CLHYO), CNR-ISTM, Via Elce di Sotto 8, Perugia I-06123, Italy; 3 Computet, Istituto Italiano di Tecnologia, Via Morego 30, 16163 Genova, Italy; 4 Laboratory for Photonics and Interfaces (LPI), Institute of Chemical Sciences and Engineering, Swiss Federal Institute of Technology, Lausanne CH-1015, Switzerland; 5 Solaronix S.A. Rue de l'Ouriette 129, Aubonne 1170, Switzerland

## Abstract

Despite the impressive photovoltaic performances with power conversion efficiency beyond 22%, perovskite solar cells are poorly stable under operation, failing by far the market requirements. Various technological approaches have been proposed to overcome the instability problem, which, while delivering appreciable incremental improvements, are still far from a market-proof solution. Here we show one-year stable perovskite devices by engineering an ultra-stable 2D/3D (HOOC(CH_2_)_4_NH_3_)_2_PbI_4_/CH_3_NH_3_PbI_3_ perovskite junction. The 2D/3D forms an exceptional gradually-organized multi-dimensional interface that yields up to 12.9% efficiency in a carbon-based architecture, and 14.6% in standard mesoporous solar cells. To demonstrate the up-scale potential of our technology, we fabricate 10 × 10 cm^2^ solar modules by a fully printable industrial-scale process, delivering 11.2% efficiency stable for >10,000 h with zero loss in performances measured under controlled standard conditions. This innovative stable and low-cost architecture will enable the timely commercialization of perovskite solar cells.

## Abstract (web summary)

Up-scaling represents a key challenge for photovoltaics based on metal halide perovskites. Using a composite of 2D and 3D perovskites in combination with a printable carbon black/graphite counter electrode; Grancini *et al*., report 11.2% efficient modules stable over 10,000 hours.

With power conversion efficiencies (PCE) beyond 22%, comparable to silicon solar cells at half of the price^1^,^2^,^3^, organo lead-halide perovskite solar cells (PSC) are leading the photovoltaic research scene. However, despite the big excitement, the unacceptably low-device stability under operative conditions currently represents an apparently unbearable barrier for their market uptake^4^,^5^. Notably, a marketable product requires a warranty for 20–25 years with <10% drop in performances. This corresponds, on standard accelerated aging tests, to having <10% drop in PCE for at least 1,000 h. Hybrid perovskite solar cells are still struggling to reach this goal. Perovskite are sensitive to water and moisture, ultraviolet light and thermal stress^6^,^7^,^8^. When exposed to moisture, the perovskite structure tend to hydrolyse^6^, undergoing irreversible degradation and decomposing back into the precursors, for example, the highly hygroscopic CH_3_NH_3_X and CH(NH_2_)_2_X salts and PbX_2_, with X=halide, a process that can be dramatically accelerated by heat, electric field and ultraviolet exposure^7^,^8^. Material instability can be controlled to a certain extent using cross-linking additives^9^ or by compositional engineering^10^, that is, adding a combination of Pb(CH_3_CO_2_)_2_·3H_2_O and PbCl_2_ in the precursors^11^ or using cation cascade, including Cs and Rb cations, as recently demonstrated^2^,^3^, to reduce the material photo-instability and/or optimize the film morphology. However, solar cell degradation is not only due by the poor stability of the perovskite layers, but can be also accelerated by the instability of the other layers of the solar cell stack. For instance, the organic hole transporting material (HTM) is unstable when in contact with water. This can be partially limited by proper device encapsulation^12^,^13^,^14^ using buffer layers between perovskite and HTM^15^ or moisture-blocking HTM^16^ such as NiO_*x*_ (ref. 17) delivering, in this case, up to 1,000 h stability at room temperature. However, this approach increases the device complexity, and the cost of materials and processing. It is also worth to mention that most of the device stability measurements reported in literature are often done under arbitrary conditions far from the required standards^18^ such as not performed under continuous light illumination^17^, measured at an undefined temperature, or leaving the device under uncontrolled light and humidity conditions^19^. This makes a proper comparison among the different strategies used challenging. On the other hand, two-dimensional (2D) perovskites have recently attracted a substantial interest due to their superior stability and water resistance, far above their three-dimensional (3D) counterpart^14^,^20^,^21^. In this respect, solar cells based on the quasi-2D (BA)_2_(MA)_2_Pb_3_I_10_ (BA=n-butylammonium) perovskite have recently shown 12% efficiency^21^. However, their performances drop by 30% after running for 2,250 h in ambient conditions.

Here we develop an innovative concept by engineering a multi-dimensional junction made of 2D/3D perovskites. This 2D/3D interface brings together the enhanced stability of 2D perovskite with the panchromatic absorption and excellent charge transport of the 3D ones, enabling the fabrication of efficient and ultra-stable solar cells, an important proof of concept for further device optimization and up-scaling. In particular, we develop HTM-free solar cells and modules substituting the HTM with hydrophobic carbon electrodes^22^,^23^. Within this configuration we demonstrate, for the first time, a remarkable long-term stability of >10,000 h, corresponding to >400 days with zero loss in efficiency over a large-area, fully printable, low-cost and high-efficient solar module of 100 cm^2^ (active area of around 50 cm^2^) measured under controlled standard conditions and in the presence of oxygen and moisture.

## Results

### Structural and optoelectronic characterization

**Figure 1:**
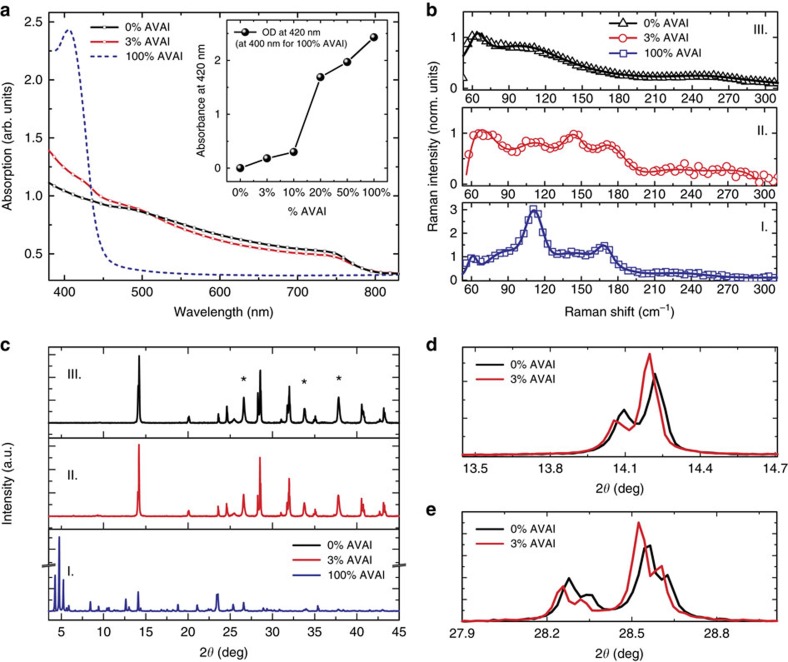
Optical and Structural characterization. (**a**) Absorption spectra of the (HOOC(CH_2_)_4_NH_3_)_2_PbI_4_ (blue dashed line), 3D CH_3_NH_3_PbI_3_ (black line) and 2D/3D (red line) using 3% of HOOC(CH_2_)_4_NH_3_I, AVAI hereafter. In the inset the intensity of the peak at 420 nm with increasing the percentage of AVAI to PbI_2_. (**b**) Raman spectra for 100%AVAI (panel I.), 3%AVAI (panel II) and 0%AVAI (panel III) perovskites. Solid lines represent the fit from multi-gaussian peaks fitting procedure (Supplementary Fig. 3 for details). For 3D perovskite main peak at: 78, 109 and 250 cm^−1^; for 2D at: 73, 109, 143, 171 cm^−1^ and for 2D/3D at: 62, 87, 112, 143, 169 cm^−1^; (**c**) X-Ray diffraction pattern of 100%AVAI (panel I); 3%AVAI (panel II) and 0%AVAI (panel III) perovskite. Peaks denoted with a star originate from the FTO/TiO_2_ substrate. (**d**) Zoom of the X-ray diffraction pattern comparing the 3%AVAI with the pure 0%AVAI perovskites at selected angles. Substrate: mesoporous TiO_2_.

**Figure 2:**
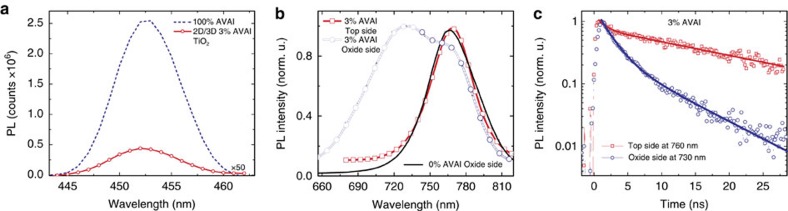
Emission Properties. (**a**) PL spectra, excitation at 400 nm, for the 100% HOOC(CH_2_)_4_NH_3_I, AVAI hereafter and 2D/3D at 3%AVAI, exciting from the TiO_2_ side, where perovskite is infiltrated within the mesoporous scaffold. (**b**) Normalized PL spectra, excitation at 600 nm, for the 2D/3D at 3% AVAI exciting from the top perovskite layer and from the TiO_2_ side, where perovskite is infiltrated within the mesoporous scaffold compared to 3D 0%AVAI exciting from the mesoporous side (solid line). Since the light penetration depth is <100 nm at 600 nm, excitation of the perovskite film from the oxide side (scaffold thickness of around 1 μm) interrogates the perovskite nano-crystallites grown within the scaffold, while excitation from the perovskite top layer probes the intrinsic properties of the bulk perovskite growing on top. (**c**) PL dynamics of the bulk perovskite (exciting from the top layer) at 760 nm and from the oxide side at 730 nm of the 3%AVAI deposited on the insulating ZrO_2_ mesoporous substrate.

Inspired by the concept of crystal engineering and supramolecular synthons in 2D layered perovskite^24^,^25^, we have first realized a low-dimensional perovskite using the protonated salt of aminovaleric acid iodide (HOOC(CH_2_)_4_NH_3_I, AVAI hereafter), as the organic precursor mixed with PbI_2_ (see Supplementary Information for details), following the procedure of the previous work of few of us^23^,^26^. The deposition results in the formation of a low-dimensional perovskite possibly arranging into a (HOOC(CH_2_)_4_NH_3_)_2_PbI_4_ structure. A yellowish film containing needle-like crystallites is formed (Supplementary Fig. 1). As shown in Fig. 1a, the absorption spectra of the film shows a clear band edge at 450 nm and an excitonic peak at 425 nm^24^,^25^. Band edge emission at 453 nm is observed in the photoluminescence (PL) spectrum (Supplementary Fig. 2). The structural properties of the (HOOC(CH_2_)_4_NH_3_)_2_PbI_4_ are investigated by Raman spectroscopy and X-ray diffraction. The Raman spectra of the 100%AVAI sample is compared with the one collected from the 3D perovskite (0%AVAI) and to the mixed 3% AVAI, as shown in Fig. 1b (panels I–III). For the 100%AVAI perovskite sharper peaks in the 50–200 cm^−1^ range are observed. More in details, the peaks at 87, 112 and 169 cm^−1^ are related to *Pb*-*I* stretching and bending modes^27^,^28^,^29^, while the modes at 62 and at 143 cm^−1^ are associated to the rotation and libration of the organic cations, leading to an overall spectra very similar in shape to that of PbI_2_ intercalated with ammonia molecules^27^,^28^,^29^. The X-ray diffraction measurement of the 100%AVAI perovskite, Fig. 1c (panel I), collected on an extended range down to 3°, exhibits a rich diffraction pattern at low angles with a strong dominant peak at 4.7° along with two lateral peaks at 4.2 and 5.2°. The data provide evidence of the formation of a low-dimensional perovskite with a possible much more complex crystal structure as evident by the multiple reflections at low angles (2*θ*<10^o^)^24^,^25^,^30^. As the second step, we engineer the 2D/3D composite by mixing the (AVAI:PbI_2_) and (CH_3_NH_3_I:PbI_2_) precursors at different molar ratios (0–3–5–10–20–50%, as described in Supplementary Information). The mixed solution is infiltrated in the mesoporous oxide scaffold by a single-step deposition followed by a slow drying-process, allowing the reorganization of the components in the film before solidification. The mixed films obtained by varying the precursors ratio absorb across the whole visible region with an edge at 760 nm and a peak around 430 nm, see Fig. 1a and Supplementary Fig. 4. Figure 1a shows the results for the film obtained by 3% AVAI, representing the optimized concentration for the best performing device (see below). The absorption band edge matches with that of 3D CH_3_NH_3_PbI_3_ perovskite, while the peak at 430 nm, which linearly gains intensity upon increasing the AVAI% (see inset in Fig. 1a and Supplementary Fig. 4), resembles the absorption peak of the 2D perovskite, although partially red-shifted. The addition of 3% AVAI thus induces the formation of a mixed 2D/3D composite, partly retaining the features of its 2D and 3D constituents. Figure 1b compares the Raman spectrum of the 100%AVAI sample with those from the 0%AVAI and the optimized 3% AVAI sample. Additional data at different AVAI concentration are reported in Supplementary Figs 5 and 6. The spectrum of the 2D/3D 3%AVAI composite shows well-defined Raman lines spectrally matching the 2D peaks and standing out of a broader band that is characteristic of the modes of the inorganic lattice of the 3D CH_3_NH_3_PbI_3_ (refs 28, 29). In the 3% AVAI sample, sharp Raman features with reduced broadening are identified, suggesting an overall more ordered crystal rearrangement of the 2D/3D film compared with the pure 3D phase. The X-ray diffraction pattern of the 2D/3D is reported in Fig. 1c–e and Supplementary Figs 7 and 8 for the different AVAI content. The most prominent peak is observed at 14.13°, related to the (110) direction of the CH_3_NH_3_PbI_3_ tetragonal phase^30^. More in details, as revealed by the zoom in Fig. 1d,e, the 2D/3D film shows a remarkable change in intensity of the (00l) and (hk0) peaks compared with the pattern of the 3D CH_3_NH_3_PbI_3_ perovskite. With 3% AVAI both (002) and (004) decrease in intensity, while the intensity of the (110) and (220) reflections are increased, speaking in favour for a preferred orientation along the <hk0> direction^23^. On the other side, no clear evidence of the peaks related to the 2D phase is observed for 3% AVAI, while they appear if the AVAI percentage exceeds 10% (Supplementary Figs 7 and 8). We can rationalize these results suggesting that the 2D/3D perovskite film with 3% AVAI is constituted by a thin layer (possibly a monolayer) of 2D perovskite; an oriented interface where the 3D phase has a marked preferential growth direction, and a pure 3D perovskite arranging in the tetragonal phase on top. To further elucidate the properties of the 2D/3D interface, we measure the steady-state PL spectra and time-resolved PL dynamics, see Fig. 2.

In particular, we measure the 2D/3D perovskite infiltrated into an inert ZrO_2_ scaffold by varying the excitation side to selectively interrogate the perovskite crystals within the oxide or the top bulk perovskite layer, Fig. 2a. The PL measured when exciting from the oxide side reveals a weak emission around 450 nm, matching with the one of (HOOC(CH_2_)_4_NH_3_)_2_PbI_4_ (Supplementary Figs 2 and 10a). This suggests the presence of a 2D phase mostly retained at the interface with the oxide due to the favourable anchoring of the carboxylic acid group of the AVAI ligand to the TiO_2_ scaffold^31^. Monitoring a more extended spectral window, Fig. 2b, excitation of the bulk top layer results in a single PL peak at 760 nm, as one would expect, while excitation from the oxide side leads to a peak at 730 nm along with a shoulder at 760 nm. The emission at 730 nm suggests that a different perovskite phase with a larger band-gap (of 1.69 eV) is formed within the oxide scaffold, but only in the presence of the AVAI precursor. Interestingly, a similar higher energy emission has been found at low-temperature, for the 3D CH_3_NH_3_PbI_3_ perovskite, but was never observed at room temperature^32^,^33^. If we deposit a thicker oxide scaffold, avoiding the formation of the bulk CH_3_NH_3_PbI_3_ perovskite capping layer, only the peak at 730 nm appears (Supplementary Figs 9, 10), confirming that the emission at 760 nm comes from the bulk perovskite. Notably, the pristine 3D CH_3_NH_3_PbI_3_ excited from the oxide side does not show such blue-shifted emission. Figure 2c compares the PL dynamics probing the temporal decay of the two different peaks. At 760 nm, the PL shows a long-lived decay (extending out of our temporal window) typically assigned to electron-hole recombination at the band edges^32^,^33^, while at 730 nm a fast component with a time constant *τ*=2 ns dominates, see Supplementary Table 1. Interestingly, such faster decay has been observed in the more oriented 3D CH_3_NH_3_PbI_3_ that appears only at low temperature^32^,^33^, possibly due to intrinsic reduced electron-hole lifetime^34^. Note that we intentionally use the insulating ZrO_2_ substrate to highlight the intrinsic behaviour of the two phases, however a relative shortening of the lifetime is also observed on TiO_2_ (Supplementary Fig. 11).

Overall, our analysis demonstrates the unique role of the 2D perovskite, anchored on the oxide network, in templating the growth of a biphasic 3D CH_3_NH_3_PbI_3_: an oriented wider band-gap phase within the oxide scaffold and a standard tetragonal phase on top of it. It is important to remark the fundamental role of the oxide in templating the graded 2D/3D interface. Indeed, if the 3% AVAI perovskite is deposited on a compact glass substrate the blue-shifted emission peak at 730 nm is not observed, while only the emission at around 760 nm is visible, independently from the excitation side (see Supplementary Figs 12,13 and the discussion in Supplementary Information).

### Simulation of the 2D/3D interface

**Figure 3:**
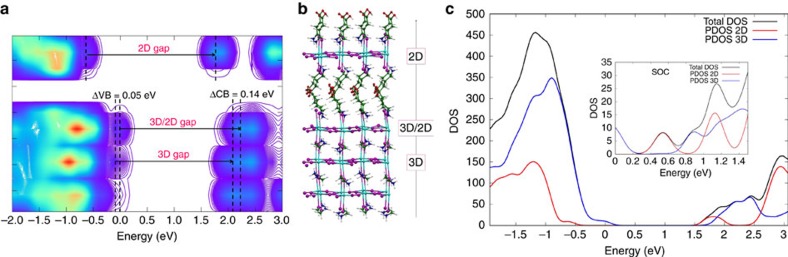
First principles simulations of the 2D/3D interface. (**a**) Local density of state (DOS) of the 3D/2D interface and (**b**) interface structure with the 2D phase contacting the TiO_2_ surface. (**c**) Partial DOS summed on the 2D and 3D fragments calculated by including spin-orbit-coupling (SOC, inset) and without it. Notice the favourable alignment of conduction band states for electron injection into the 2D perovskite and possibly further into TiO_2_.

To check the impact of the suggested 2D/3D perovskite interface on the composite electronic properties, we carried out first principles simulations of the 2D/3D interface, Fig. 3. We built periodic (in the direction orthogonal to the interface) slabs of the 2D and 3D perovskites ensuring a lattice mismatch within <1%. The methylammonium cations of the 3D perovskite layer contacting the 2D slab were replaced by AVA cations from the 2D slab and, employing the cell parameters of the 3D perovskite, we relaxed the atomic positions of the overall system by means of scalar-relativistic plane-wave/pseudopotential density functional theory calculations employing the PBE functional. As seen in Fig. 3a, there is a 0.14 eV CB upshift at the 2D/3D interface compared to the bulk of the 3D perovskite, which induces a 0.09 eV larger interface gap compared to the 3D bulk. This is clearly consistent with the PL blue shift experimentally observed (0.13 eV) when probing the system from the oxide side. Notably, only a small shift (around 0.02 eV) of opposite sign was found at the MAPbI_3_/TiO_2_ interface^35^.

## Discussion

These results suggest that the 2D/3D interaction widens the 3D perovskite band-gap in the interface region. Additionally, the thin 2D layer does not constitute a barrier to electron injection to TiO_2_, but it rather constitutes a barrier towards electron recombination, since the 2D conduction band is found at lower energy than that of the 3D CB. This result is confirmed also in the presence of spin-orbit-coupling, see PDOS in Fig. 3c and Supplementary Fig. 14. The results indicate that the 2D/3D perovskite organizes in a gradual multi-dimensional structure retaining the individual 2D and 3D phases, but, importantly also templating the formation of a novel oriented CH_3_NH_3_PbI_3_ phase stabilized at the 2D/3D interface.

**Figure 4:**
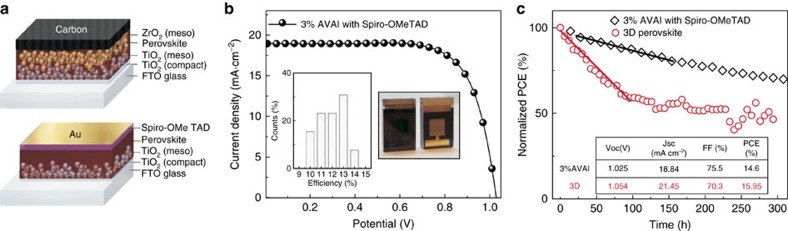
2D/3D Mesoporous Solar cell characteristics and stability. (**a**) Device cartoon of the Hole transporting Material (HTM)-free solar cell and of the standard HTM-based solar cell. (**b**) Current density voltage (*J*–*V*) curve using the 2D/3D perovskite with 3% HOOC(CH_2_)_4_NH_3_I, AVAI hereafter, in a standard mesoporous configuration using 2,2′,7,7′-tetrakis(N,N-di-p-methoxyphenylamine)-9,9′-spirobifluorene (spiro-OMeTAD)/Au (devise statistics and picture of the cell in the inset). (**c**) Stability curve of the Spiro-OMeTAD/Au cell comparing standard 3D with the mixed 2D/3D perovskite at maximum power point under AM 1.5G illumination, argon atmosphere and stabilized temperature of 45 °C. Solid line represent the linear fit. In the inset the champion device parameters are listed.

We have then fabricated solar cells with the 2D/3D perovskite optimizing the AVAI% using both an architecture with an organic HTM and Au electrode, and a fully printable HTM-free configuration, where the HTM and gold are substituted with a carbon matrix^23^, and a TiO_2_ mesoporous layer as electron transporting layer^36^, as depicted in the cartoon in Fig. 4a. *J*–*V* characteristic of the 2D/3D perovskite solar cell using an optimal 3%AVAI composition and Spiro-OMeTAD/Au is shown in Fig. 4b. The solar cell delivers a champion efficiency of 14.6% (see also Table in the inset of Fig. 4c, Supplementary Fig. 15 and device statistics in the inset and in Supplementary Figs 16,17). Importantly, it shows a much better trend in the device stability with respect to pure 3D CH_3_NH_3_PbI_3_ cell (delivering an average efficiency above 13% in the same cell architecture), as shown in Fig. 4c. This represents an important proof of concept that paves the way to further stabilize the high efficiency (beyond 21%) mesoporous solar cells based on a mixed halide composition^2^,^37^,^38^. Using the 2D/3D perovskite compared to the standard 3D CH_3_NH_3_PbI_3_, the efficiency is maintained up to 60% of the initial value after 300 h continuous illumination under argon atmosphere, more stable than the standard 3D perovskite (Fig. 4c).

**Figure 5:**
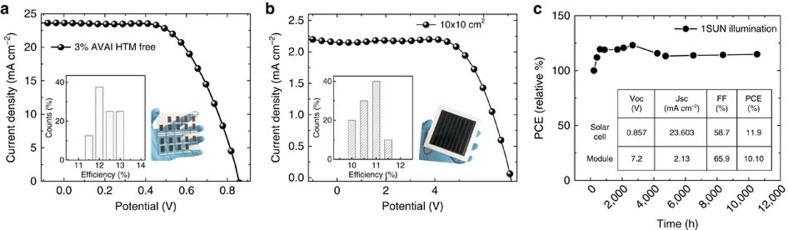
2D/3D Carbon based Solar cell characteristics and stability. (**a**) *J*–*V* curve using the 2D/3D perovskite with 3%AVAI in HTM-free solar cell measured under Air Mass (AM) 1.5G illumination (device statistics and picture in the inset). (**b**) *J*–*V* curve using the 2D/3D perovskite with 3%AVAI in a HTM-free 10 × 10 cm^2^ module (device statistics and picture in the inset). (**c**) Typical module stability test under 1 sun AM 1.5 G conditions at stabilized temperature of 55° and at short circuit conditions. Stability measurements done according to the standard aging conditions. In the inset device parameters of the devices represented in **a** and **b**. Champions devices reported in Supplementary Table 2.

On the other side, based on the pioneering work by Mei *et al*.^23^ we developed HTM-free solar cells, being at present the cheapest, fully printable low-cost deposition process. It presents the most attractive photovoltaic solution, being a simple monolithic architecture without the use of the instable and expensive organic HTM and without the additional barrier layers^17^. We have developed small area cells (0.64 cm^2^) and large area 10 × 10 cm^2^ solar modules as shown in Fig. 5a,b reporting the average *J–V* curve of the cell and the module, as well as the device statistics in the inset. The modules were prepared with a total size of 10 × 10 cm^2^, with a geometric fill factor (GFF), the ratio between the active area and the total area of the module, of 46.7%. This leads to an active area of 47.6 cm^2^ per module, which is the area considered to calculate the device efficiency. In addition, to avoid any mechanical damage of the cells, the devices were protected with a glass slide via a very simple sealing method under air conditions (see details in Supplementary Information), not under any inert or humidity controlled atmosphere as usually reported in literature, further confirming the higher robustness of our devices. The champion cell and module deliver an efficiency of 12.71% and 11.2%, respectively, among the highest reported so far ranging from 7 to 14% (refs 39, 40, 41, 42), as shown in Supplementary Table 2 (ref. 22). It is worth mentioning that a fair comparison among the majority of reported module efficiencies is challenging because the GFF and the geometric shape of the devices are most of the times not indicated. In our work, the modules consist of 8 cells of 85 × 7 mm^2^, which leads to an active area of 5.95 cm^2^ per cell and 47.60 cm^2^ per module. In this module design, both the interconnect distance (around 3 mm) and the margins around the module aperture are admittedly large, implying an important loss in the area (which is included in the total area considered for the module). Further optimizations could be done by reducing the interconnect distance between cells, which will probably have two different impacts: less area will be lost, increasing the efficiency per total area in the module and the ohmic losses at the FTO between the interconnect gap will be reduced, improving the efficiency per active area. In addition, producing larger modules will not significantly increase the area for the margins, which would also have a positive impact on the final module performance. We have tested the modules under different conditions under simulated AM 1.5 G solar illumination at 1000 W m^−2^ and cycling of temperature up to 90 °C under ambient conditions, in agreements with the standards (Supplementary Fig. 17). The results, in Fig. 5c, show an extraordinary long-term stability of >10,000 h and excellent response at elevated temperature (Supplementary Fig. 17), reported here for the first time. It is fair to notice that an initial increase is detected in Fig. 5c in the first 500 h of the stability test. This can be due to concomitant effects such as light or field induced ion movement with the associated structural rearrangement, light-induced trap formation, or interfacial charge accumulation that can alter the device behaviour (and also cause the device hysteresis), at present under intense scrutiny^37^,^43^,^44^. Supplementary Fig. 18 and Supplementary Table 4 report the comparison of the *J*–*V* characteristic and parameters, when the device is measured in forward and back scan direction. As mentioned above, it is fair noticing that these devices show a not negligible hysteresis that is subject of ongoing investigation. Remarkably, the long stability here reported is at present the highest record value obtained for perovskite photovoltaics, surpassing with a gigantic step the results obtained so far.

In conclusion, we interface engineering a built-in 2D/3D perovskite which grows forming a peculiar bottom-up phase-segregated graded structure. The unique combination of the 2D layer acting as a protective window against moisture, preserving the 3D perovskite and of the efficient 3D one provides the hint for the development of a stable perovskite technology, paving the way for the realization of near term high efficient and stable perovskite solar cells for widespread deployment.

## Methods

### Materials development

CH_3_NH_3_I (MAI), HOOC(CH_2_)_4_NH_3_I (AVAI) and TiO_2_ paste (30 NR-D) have been purchased from Dyesol Company. PbI_2_ was purchased from TCI Europe. All chemicals were used as received without further purification.

### Fabrication of Spiro-OMeTAD based solar cells

The solar cells were prepared on fluorine-doped tin oxide coated glass (NSG10) substrates. Before the deposition of the different layers, the substrates were cleaned by sequentially sonicating them during 15 min in hellmanex solution (2 vol%), 5 min in distilled water and finally 5 min more in isopropanol, followed by 15 min of ultraviolet-ozone treatment. A 30 nm thick TiO_2_ blocking layer was deposited by spray pyrolysis from a solution containing 600 μl Titanium diisopropoxide bis(acetylacetonate) 75% from Sigma Aldrich in 9 ml of isopropanol. After sintering at 450 °C, the mesoporous TiO_2_ layer was spin-coated from a 400 mg ml^−1^ solution of 30NRD Dyesol paste in EtOH at 1,000 r.p.m. for 10 s and annealed at 500 °C for 30 min. Once the substrates were cooled down, 40 μl of a solution containing 1.25 M PbI_2_ /MAI (1:1) in DMSO was spin-coated on top through a two-step process: the first step consisting of 1,000 r.p.m. during 10 s (preconditioning of the layer) and the second step at 4,500 r.p.m. during 30 s. Ten seconds before the end of the program, 100 ml of chlorobenzene were spin-coated on top of the perovskite layer, according to the antisolvent method previously described in literature^38^, and finally the perovskite films were sintered at 100 °C during 1 h. Different compositions of 2D–3D perovskite were also tested by mixing different amounts of 1.1 M solution of AVAI:PbI_2_ (2:1) with the MAI:PbI_2_ precursor solution (0, 3 and 5% molar ratio). After the annealing time, Spiro-OMeTAD was spin-coated at 4,000 r.p.m., 20 s from a chlorobenzene solution (28.9 mg in 400 μl, 60 mmol) containing Li-TFSI (7.0 μl from a 520 mg ml^−1^ stock solution in acetonitrile), TBP (11.5 μl) and Co(II)TFSI (10 mol%, 8.8 μl from a 40 mg ml^−1^ stock solution) as dopants. Finally, a 100 nm gold electrode was evaporated.

### Fabrication of carbon-based mesoscopic solar cells

The FTO glass was first etched to form two separated electrodes before being cleaned ultrasonically with ethanol. Then, the patterned substrates were coated by a compact TiO_2_ layer by aerosol spray pyrolysis, and a 1 μm nanoporous TiO_2_ layer was deposited by screen-printing of a TiO_2_ slurry, which was prepared as reported previously^23^. After being sintered at 450 °C for 30 min, a 2 μm ZrO_2_ spacer layer was printed on the top of the nanoporous TiO_2_ layer using a ZrO_2_ slurry, which acts as an insulating layer to prevent electrons from reaching the back contact. Finally, a carbon black/graphite counter electrode with a thickness of about 10 μm was coated on top of the ZrO_2_ layer by printing a carbon black/graphite composite slurry, and sintering at 400 °C for 30 min. After cooling down to room temperature, the perovskite precursor solution was infiltrated through a semi-continuous printing process from the top of the carbon counter electrode by drop casting. The complete printing process was carried out in air conditions. After drying at 50 °C for 1 h, the mesoscopic solar cells containing perovskite was obtained. The perovskite precursor solution was prepared as follows: for the 3D precursor solution, 1.2 M of MAI and 1.2 M of PbI_2_ were dissolved in γ-butyrolactone, and then stirred at 60 °C overnight. For the 2D perovskite 1.2 M of AVAI and 1.2 M of PbI_2_ were dissolved in γ-butyrolactone and then stirred at 60 °C overnight. The (AVA)_*x*_(MA)1-xPbI_3_ precursor solution was prepared in the same manner except that a mixture of (AVAI:PbI_2_) and (MAI:PbI_2_) with 3, 10, 20, 50 vol% (that is, (AVAI:PbI_2_)/((AVAI:PbI_2_)+(MAI:PbI_2_))) was used. All the cells were encapsulated in ambient atmosphere to protect the cell from mechanical damage, with no special control on the humidity and oxygen content. The encapsulation was performed by covering the cells with a thin glass and sealing the edges using DuPont Surlyn polymer. In the case of the modules, the same process was carried out but adding an extra ring of epoxy glue around the cell as a second protection.

### X-ray diffraction measurements

X-ray diffraction measurements were done on thin films using a D8 Advance diffractometer from Bruker (Bragg–Brentano geometry). Perovskite layers grown on top of mesoporous titania, as well as mesoporous zirconia and were analysed on addition of various amounts of AVAI.

### Solar cells and module characterization

For Spiro-based solar cells, photocurrent density voltage (*J–V*) curves were characterized with a Keithley 2400 source/metre and a Newport solar simulator (model 91192) giving light with AM 1.5G spectral distribution. A black mask with an aperture (0.16 cm^2^) smaller than the active area of the square solar cell (0.5 cm^2^) was applied on top of the cell. The measured *J*_sc_ did not change using a mask.

Cells with HTM-free (0.64 cm^2^ large) were measured in air, but sealed with the glass slide and Surlyn polymer, the cell temperature during the measurements reaches 60°. Current–voltage characteristics of cells were measured under AM 1.5 simulated sunlight (class AAA solar simulator from Newport equipped with a 1000W Xenon lamp) with a potentiostat (Keithley). The light intensity was measured for calibration with an NREL certified KG5 filtered Si reference diode. Light source used is a solar simulator equipped with a discharge Xe lamp, properly calibrated using a reference Si solar cell. Same cell are also measured with an in-house developed AAA class simulator using a plasma lamp with a spectrum that exactly superimposes to the standard. No preconditioning protocol is used. Each cell is measured five times and the last one is taken as defined protocol, no differences are observed, no average of the scans are made. The module has been sealed with a back glass sheet and the stability tests were carried out in ambient air. The HTM-free cells used an adhesive mask with square aperture which is 8 × 8 mm^2^ in aperture to avoid external rays. For the module no mask is used, as the active area is the size of the glass. The total active area for each 10 × 10 cm^2^ module is 46.7 cm^2^. The overall GFF for the module that is the ratio between the active area and the total area of the module is then 46.7%, standing within the range observed for fully printed organic photovoltaic modules.

### Solar cells reproducibility

Spiro-OMeTAD solar cells: batch of 16 devices has been produced twice. HTM-free: 1 batch is 10 × 10 glass with 18 cell, in total 9 batch have been continuously produced so far. For the modules, >10 at paper submission time, 20 so far. Data are reproducible comparing batch to batch.

### Stability measurements

#### 

*Spiro-OMeTAD based solar cells.* The cells are placed in a sealed cell holder with a glass cover that is flushed with a flow of argon of 30 ml min^−1^. The holder is therefore exempt of water and oxygen, avoiding the need of sealing and improving the reproducibility. IV curves were characterized by an electronic system using 22 bits delta-sigma analogic to digital converter. For IV curves measurement, a scan rate of 25 mV s^−1^ with a step of 5 mV was used, maintaining the temperature of the holder to 35 °C while the temperature of the cells was measured around 45 °C. The system comprises a set of *I*–*V* curves at different light intensities (dark current, 10 and 100 mW cm^−2^). Between each measurement the cells are maintained at the maximum power point using a MPPT algorithm under 100 mW cm^−2^. A reference Si-photodiode is placed in the holder to verify the stability of the light.

*Carbon-based mesoscopic solar cells*. The cells are prepared and sealed with a glass cover under ambient atmosphere as previously described. For the stability the cells are at 55° temperature, 1sun illumination for 24 h per day, sealed under ambient atmosphere. An ultraviolet filter up to 390 nm is on top of all over the samples. Solar simulator class A 1.5 M at full sun under short circuit condition. Stability measurements done according to the ISOS standard conditions.

### Sample for spectroscopy

The FTO glass was first etched to form two separated electrodes before being cleaned ultrasonically with ethanol. The patterned substrates were coated by a compact TiO_2_ layer by aerosol spray pyrolysis, and 1 μm nanoporous TiO_2_ or ZrO_2_ layer were deposited by screen-printing and sintered at 450 °C for 30 min, as described previously. After cooling down to room temperature, the perovskite precursor solution was infiltrated by drop casting and let it drying at 50 °C for 1 h. The perovskite infiltrates in the mesoporous scaffold forming a capping layer of around 1 μm thick. All the samples were encapsulated with a microscope glass to prevent any interaction with oxygen and moisture.

### Absorption and photoluminescence

The absorption spectra have been registered with a ultraviolet–vis-infrared spectrophotometer (PerkinElmer Instrument). Photoluminescence (PL) Measurements: CW and time-resolved PL experiments were performed with a spectrophotometer (Gilden Photonics) using the lamp or a pulsed source at 460 nm (Ps diode lasers BDS-SM, pulse with<100 ps, from Photonic Solutions, 20 MHz repetition rate, ∼500 μm spot radius), respectively. The excitation density is around few nJ cm^−2^. The steady-state spectra and the time-resolved signal were recorded by a photomultiplier tube, and by a Time Correlated Single Photon Counting detection technique with a time resolution of 1 ns, respectively. A monoexponential and bi-exponential fitting were used to analyse the background-corrected PL decay signal.

### Raman spectroscopy

The micro-Raman system is based on an optical microscope (Renishaw microscope, equipped with × 5, × 20, × 50 and × 100 short and long working distance microscope objectives) used to focus the excitation light and collect it in a back scattering configuration, a monochromator, notch filters system and a charge coupled detector. The sample is mounted on a translation stage of a Leica microscope. The excitation used consists of a laser diode at 532 nm. The system has been calibrated against the 520.5 cm^−1^ line of an internal silicon wafer. The spectra have been registered in the 50–250 cm^−1^ range, particularly sensitive the Pb-I modes. The final data have been averaged over 50 accumulations in order to maximize the signal to noise ratio. The measurements were conducted at room temperature on encapsulated samples using the × 100 long working distance objective. To prevent sample degradation or thermal effects the laser power intensity is kept below 50 μW.

### Computational details

DFT calculations within periodic boundary conditions have been performed within the planewave/pseudopotential formalism, as implemented in the PWSCF package of Quantum-Espresso^45^. For the geometry optimization we used the PBE exchange-correlation functional^46^ along with ultrasoft^47^, scalar relativistic pseudopotentials for all the atoms. Electrons from I 5s, 5p; O, N and C 2s, 2p; H 1s; Pb 6s, 6p, 5d shells explicitly included in the calculations. Spin-orbit coupling was included in the calculations of the DOS of Fig. 3.

The cutoffs for the wave function and the electronic density expansions were set to 25 and 200 Ry cutoffs, respectively.

Our model system for the 3D system is made by I-terminated MAPbI_3_ 2 × 2 × 3 tetragonal perovskite slab exposing the 001 surfaces. The 2D slab was obtained by using the experimental X-ray data reported for (HOOC(CH_2_)_3_NH_3_)PbI_4_ in ref. 25 To model the 2D/3D interface we deposit the 2 × 2 × 1 2D perovskite slab exposing the 001 surfaces onto the 3D slab, replacing one MA+ layers from the top of the 3D slab with one HOOC(CH_2_)_3_NH_3_ layer, see Supplementary Fig. 12.

The experimental MAPbI_3_ cell parameters (*a*=*b*=8.8556) are employed to build a periodic supercell in the *x* and *y* directions of twice the unit cell size (*a*=*b*=17.7112), leaving 10 Å vacuum along the *z* direction.

### Data availability

The data that support the findings of this study are available from the corresponding author upon request.

## Additional information

**How to cite this article:** Grancini, G. *et al*. One-Year stable perovskite solar cells by 2D/3D interface engineering. *Nat. Commun.* **8**, 15684 doi: 10.1038/ncomms15684 (2017).

**Publisher's note**: Springer Nature remains neutral with regard to jurisdictional claims in published maps and institutional affiliations.

## Supplementary Material

Supplementary Information Supplementary Figures and Supplementary Tables
