# Supplementary material for: One-Year stable perovskite solar cells by 2D/3D interface engineering
Source: Nat Commun. 2017 Jun 1;8:15684. doi: 10.1038/ncomms15684 (PMC5461484; doi:10.1038/ncomms15684)
Supplement: Supplementary Information — Supplementary Figures and Supplementary Tables [file ncomms15684-s1.pdf]

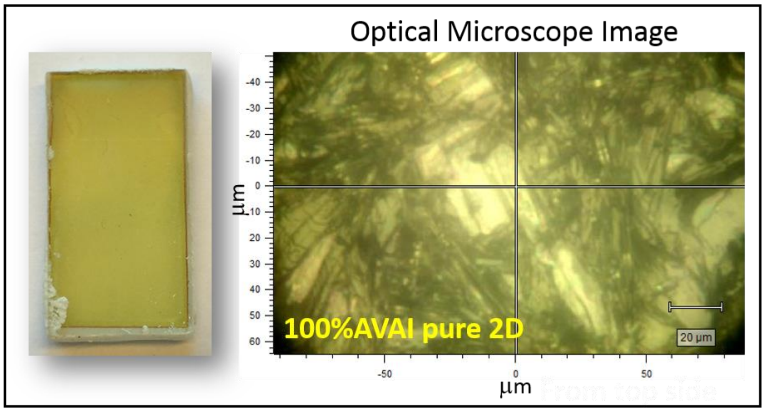

Supplementary Figure 1. (Left) Picture of the film of $(HOOC(CH_2)_4NH_3)_2PbI_4$ deposited on TiO_2. (Right) Optical image of the film. Flat lamellae and needle-like structures are observable.

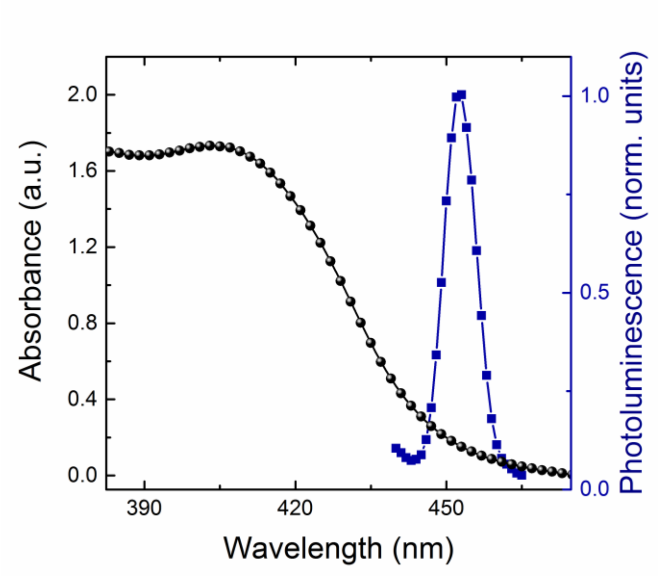

Supplementary Figure 2. Absorption and Photoluminescence spectra of the 2D $(HOOC(CH_2)_4NH_3)_2PbI_4$ deposited on TiO_2.

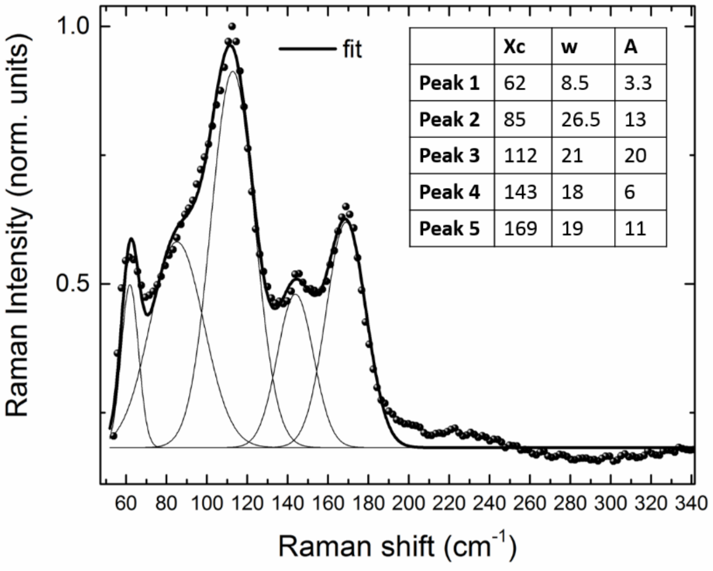

Supplementary Figure 3. Raman spectrum of the $(HOOC(CH_2)_4NH_3)_2PbI_4$ deposited on TiO_2. The solid line represent the cumulative peak fit, obtained from the sum of the single peaks as shown in the Figure. Parameters with the peak center, width and amplitude are reported in the inset. Sample is encapsulated to prevent any oxygen/moisture effect.

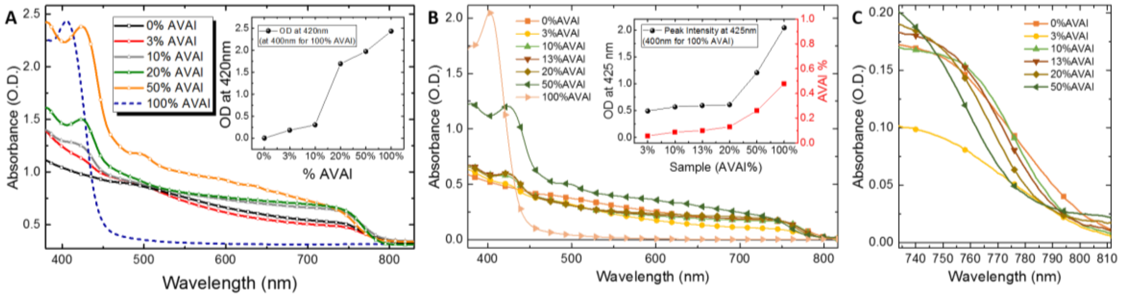

Supplementary Figure 4. a. Zoom of the absorption spectra for the series of mixed perovskite from 3% to 50% of $(AVAI:PbI_2)$/ $[(AVAI:PbI_2) + (MAI:PbI_2)]$. **b.** Amplitude of the peak at 420 nm as a function of the % of $(AVAI:PbI_2)$ in the total solution. **c.** Peak position energy as a function of the % of $(AVAI:PbI_2)$ in the total solution.

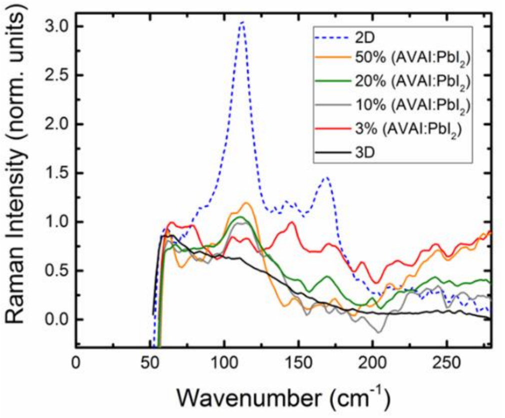

Supplementary Figure 5. Raman spectrum of the 2D $(HOOC(CH_2)_4NH_3)_2PbI_4$ and mixed 2D/3D with different % of AVAI as in the legend. Substrate: TiO_2. Samples are encapsulated to prevent any oxygen/moisture effect.

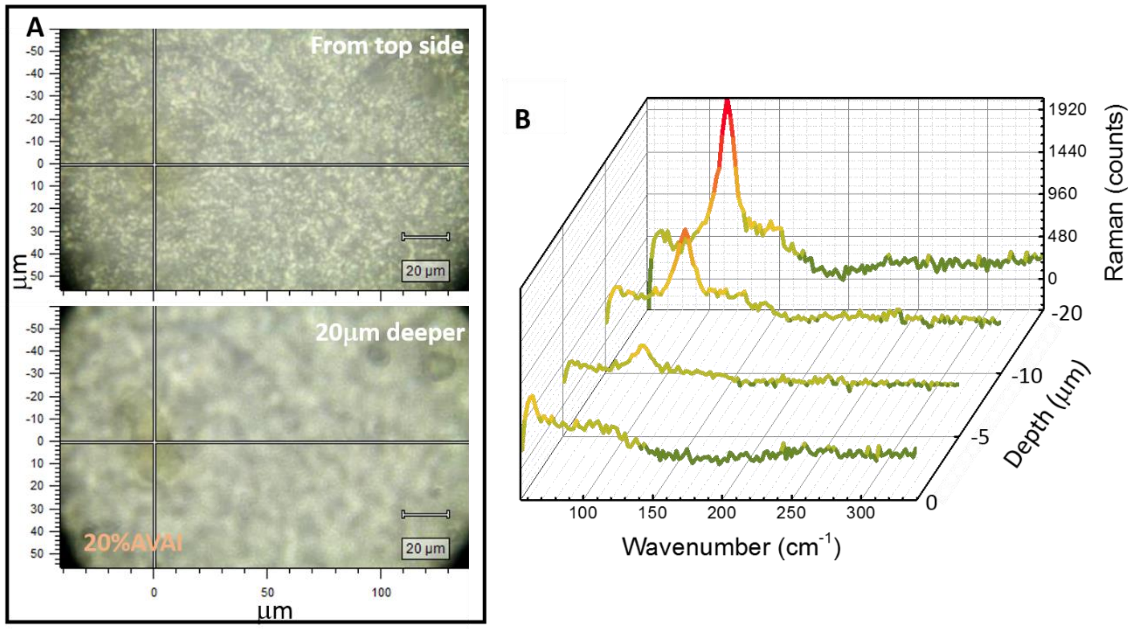

Supplementary Figure 6. a. (Top) optical microscope image of the 20% AVAI film at top film surface and (bottom) using a long working distance objective of the bottom surface 20 microns deeper. The top surface shows the perovskite grains, similarly to the 3D

case. Going deeper in the film yellowish flakes of 2D $(HOOC(CH_2)_4NH_3)_2PbI_4$ perovskite are visible, indicating that the 2D perovskite forms below the top layer, in closer proximity to the TiO_2 substrate. **b.** Raman spectrum of the 20% AVAI mixed 2D/3D at different depth from the top surface (0) deeper in the film. Going deeper the spectrum of the 2D $(HOOC(CH_2)_4NH_3)_2PbI_4$ perovskite emerges, as a clear proof that it forms closer to the TiO_2 substrate. Substrate: TiO_2. Samples are encapsulated to prevent any oxygen/moisture effect.

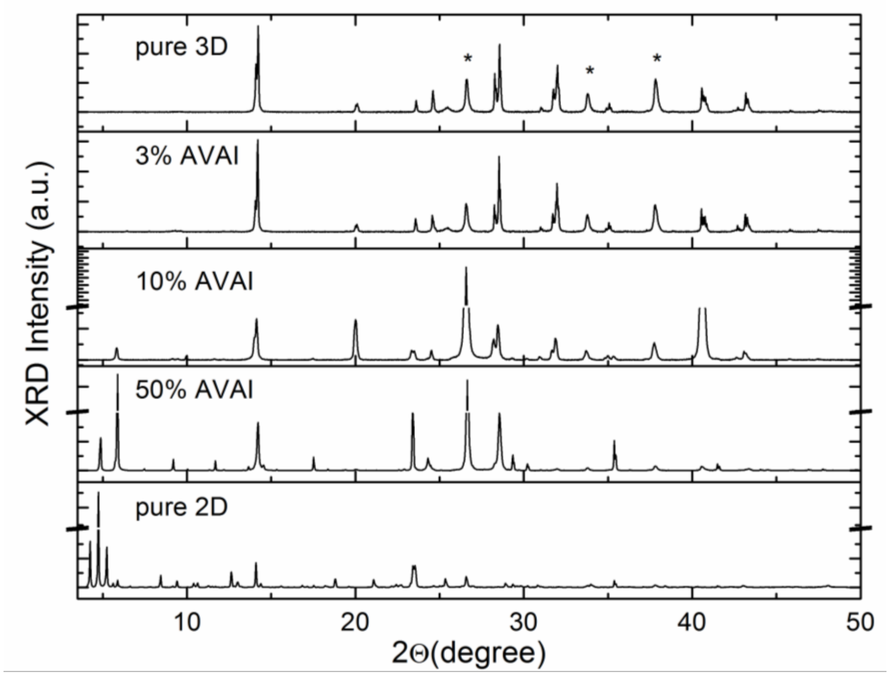

Supplementary Figure 7. XRD spectra as a function of the % of ($AVAI:PbI_2$) as in the legend. Film deposited on TiO_2 substrate.

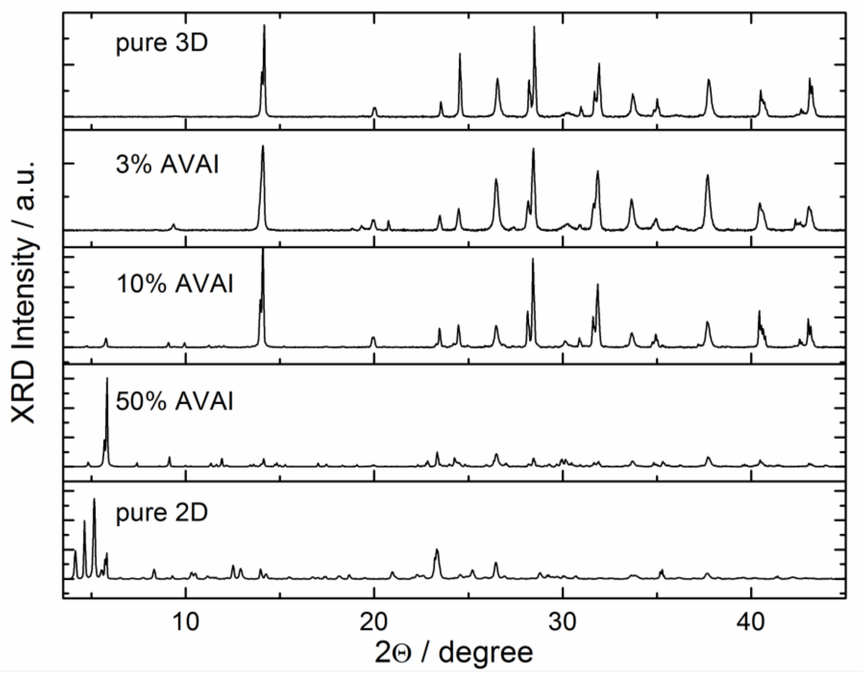

Supplementary Figure 8. XRD spectra as a function of the % of (AVAI:PbI_2) as in the legend. Film deposited on ZrO_2 substrate.

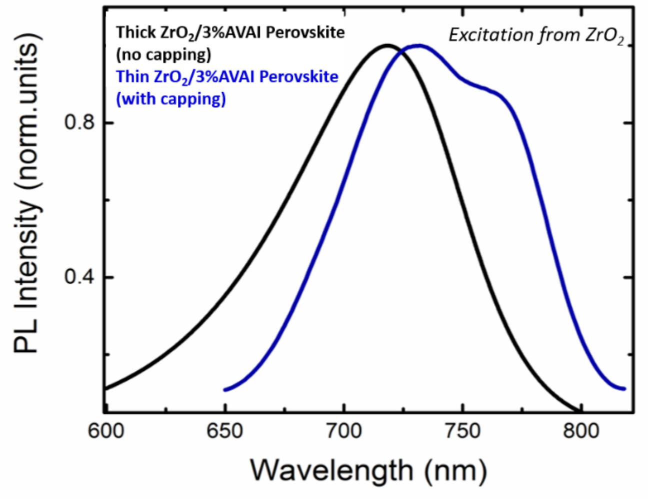

Supplementary Figure 9. PL spectra comparison of ZrO_2 /perovskite with and without the capping layer upon excitation at 600 nm from the ZrO_2 side in both cases. The

sample with no capping layer shows the only presence of the blue-shifted PL peak associated to the formation within the pores to the ordered perovskite phase.

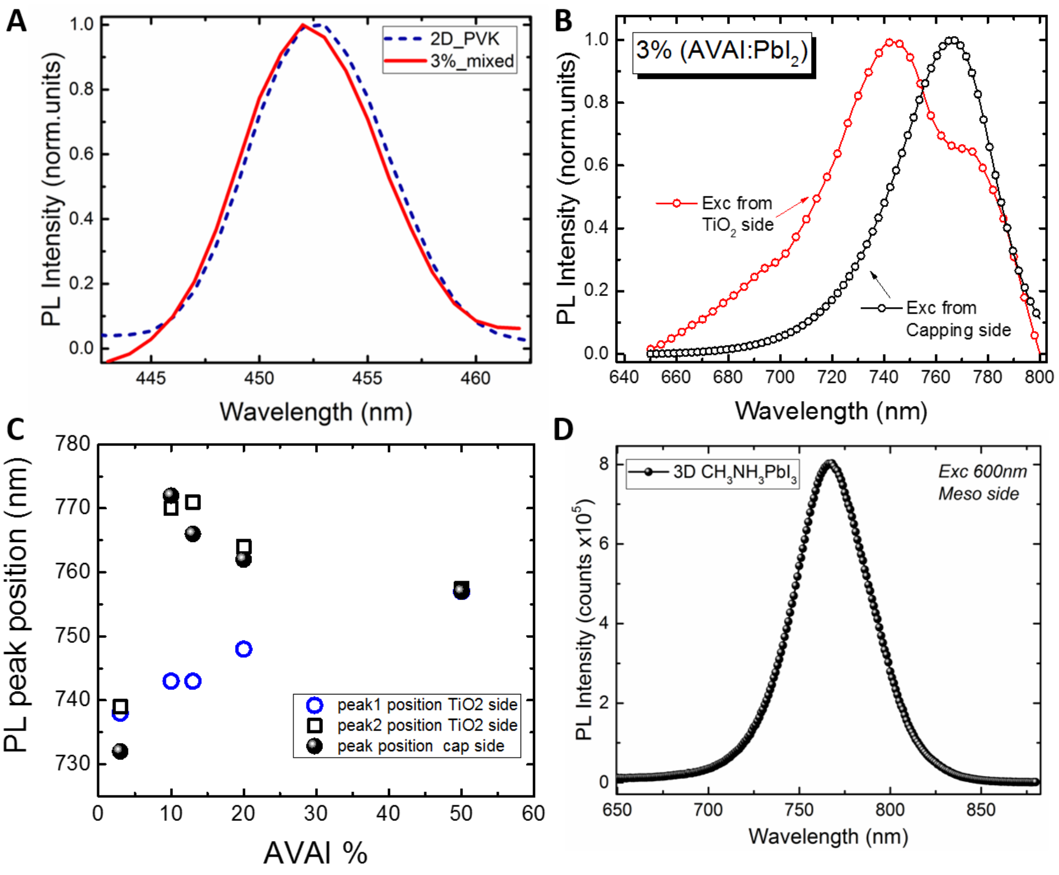

Supplementary Figure 10. a. Normalized PL spectra of $(HOOC(CH_2)_4NH_3)_2PbI_4$ perovskite compared to the 3% (AVAI:PbI_2) mixed perovskite in the TiO_2 mesoporous oxide exciting from the TiO_2 side and from the capping side. **b.** Normalized PL spectra comparison of 3%AVAI sample on TiO_2 upon excitation at 600 nm from the TiO_2 side or from the top-capping layer side. Again two peaks emerge, at around 740nm and 770nm only when exciting from the TiO_2 side. **c.** PL peak position as a function of the % of (AVAI:PbI_2) for the perovskite growth within the mesoporous TiO_2 exciting from TiO_2 and from the

capping. We observe two peaks, the blue one below 750 nm and the red shifted one above 760nm that spectrally matches with the only PL peak observed when exciting from the capping side (filled dots). **d.** For comparison, PL spectra of pristine 3D perovskite upon exciting at 600nm from the mesoporous TiO_2 side at room temperature.

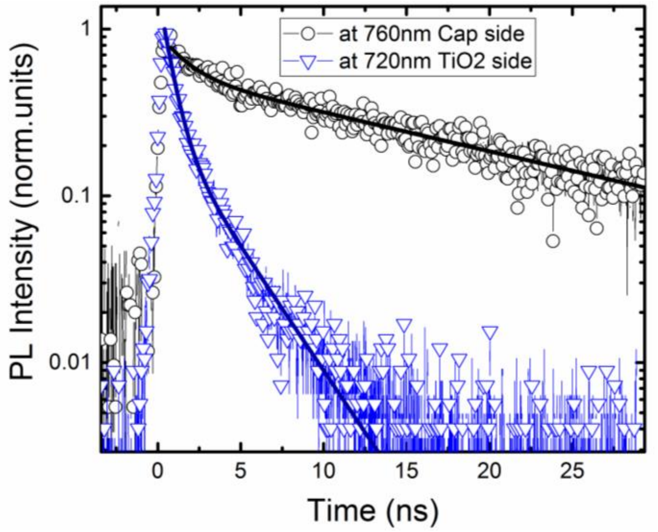

Supplementary Figure 11. PL dynamics of the "bulk" perovskite (exciting from the top layer) at 760 nm and from the oxide side at 720 nm of the 2D/3D deposited on the TiO_2 mesoporous substrate (time constants resulted from fitting are reported in Supplementary Table 1).

Supplementary Table 1.

Time constant and amplitude retrieved by fitting the PL decay shown in Fig. 2C with a bi-exponential function of the form: $y=A_1exp(-x/t)+A_2exp(-x/t)$.

		τ_1 (ns)	τ_2 (ns)	A_1 (%)	A_2 (%)
ZrO_2/mixed perovskite	ZrO_2 side at 730 nm	2	8	0.8	0.2
ZrO_2/mixed perovskite	Capping perovskite side at 760 nm	1.5	18	0.4	0.6
**TiO2/PVK**	TiO2 side at **720 nm**	**<1**	**3**	**0.85**	**0.15**
**TiO2/PVK**	PVK side at **760 nm**	1.5	18	0.44	0.56

We fabricated 3% $(AVA)_2PbI_4$ perovskite films depositing the solution on flat glass substrates to further examine the role of the mesoporous oxide in templating the graded 2D/3D interface. Results are reported in the Supplementary Figures 12 and 13.

The PL emission in Supplementary Figure 12 shows that the 3%$(AVA)_2PbI_4$ exhibits a single defined peak at around 775 nm, independently from the excitation side (see Supplementary Figure 13).

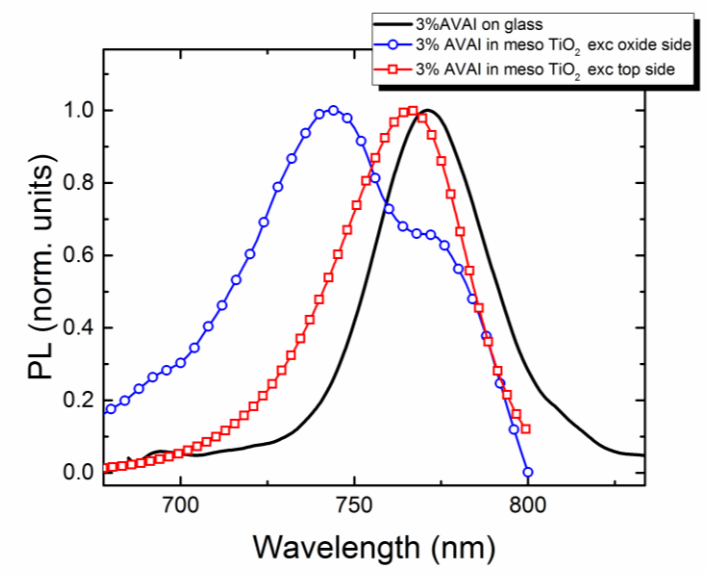

Supplementary Figure 12. Comparison of the PL spectra of the 3%$(AVA)_2PbI_4$ sample on glass (black line) to the 3% AVAI sample in the oxide scaffold excited from the top or the oxide side (as represented in Fig. 2b).

This peak corresponds to what observed when the film is deposited into the oxide scaffold upon excitation from the top layer (with a small red-shift). No blue- shifted emission is observed in this case neither a double peak emission. This result, shown in Supplementary Figure 12, suggest that the graded 2D/3D interface is not formed in this case, in contrast to what happens when the film grows in the mesoporous oxide scaffold. As we argued, the oxide scaffold has a templating role that sustains the formation of the graded 2D/3D interface.

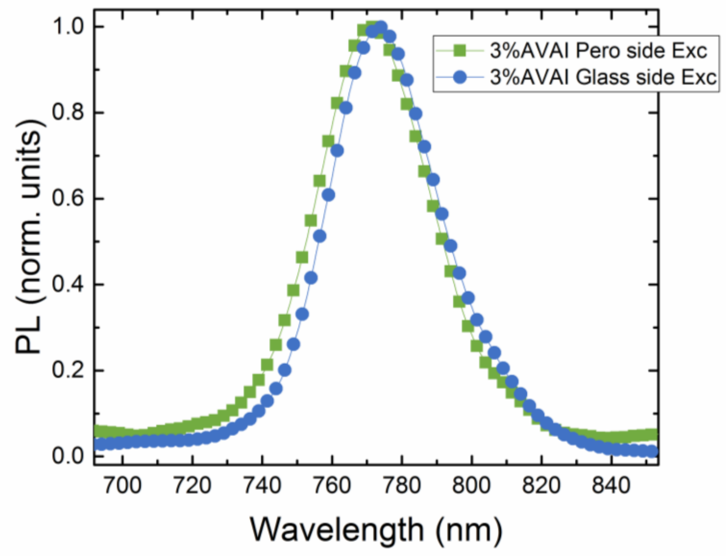

Supplementary Figure 13. PL of the 3%$(AVA)_2PbI_4$ sample upon excitation from the top side and from the bottom glass side.

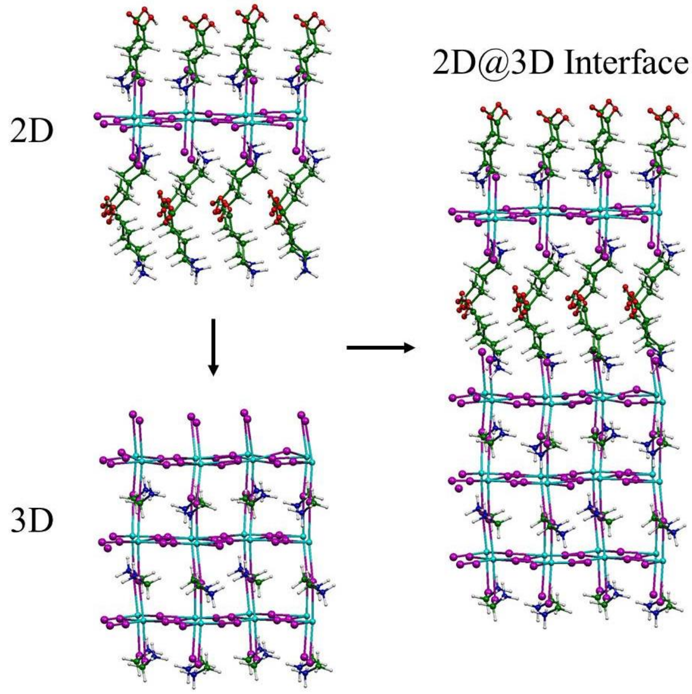

Supplementary Figure 14. Schematic representation of building the 2D/3D interface.

Supplementary Table 2. Typical photovoltaic performance of the hole conductor free solar cells exhibited by laboratory cells and modules.

	**Voc (V)**	**FF**	**Jsc (mA/cm^2)**	**Eff. (%)**
**Cell 1**	0.850	0.64	21.78	11.86
**Cell 2**	0.850	0.60	23.16	11.77
**Cell 4**	0.840	0.63	23.99	12.71

	**Voc (V)**	**FF**	**Jsc (mA/cm^2)**	**Eff. (%)**
**Module 1**	6.8	0.66	2.247	10.135
**Module 2**	6.8	0.66	2.311	10.432
**Module 3**	7.26	0.734	2.022	10.777
**Module 4**	6.72	0.55	2.73	10.156
**Module 5**	7.05	0.704	2.247	11.164

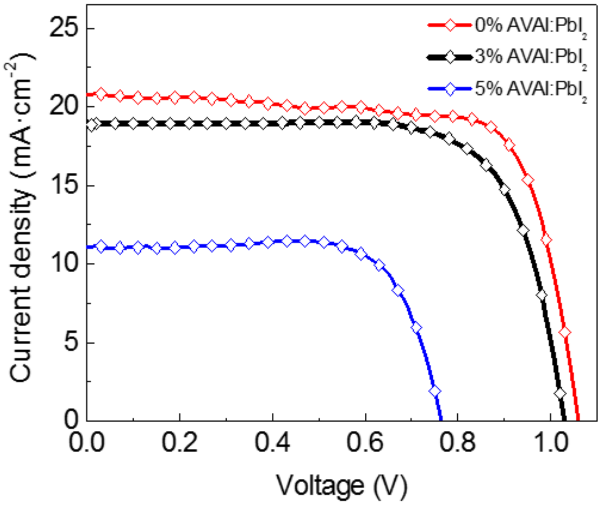

Supplementary Figure 15. J-V curve of the mixed perovskite solar cell in the mesoporous configuration changing the content of the (AVAI:PbI_2). Note that with 3% the device performances are much better than with 5%. Parameters are reported in Supplementary Table 3.

Supplementary Table 3. Device Parameter comparing back and forward measurement.

	J_{sc} (mA·cm^2)	***V_{oc} (V)***	***FF***	***PCE (%)***
**0% AVAI**	20.76	1.062	0.744	16.41
**3%AVAI**	18.84	1.025	0.755	14.6
**5% AVAI**	11.06	0.763	0.747	6.30

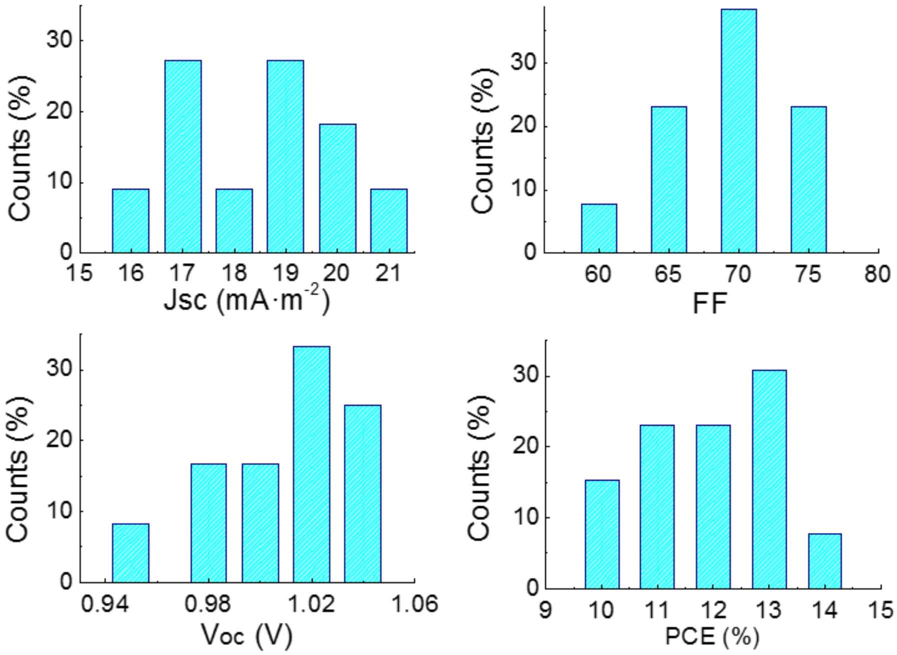

Supplementary Figure 16. Histogram of the most important parameters obtained under AM 1.5G illumination in ambient atmosphere for devices containing mixed 3% (AVAI:PbI_2) perovskite in the FTO/TiO_2(b)/TiO_2(m)/mixed 3% (AVAI:PbI_2)/Spiro-OMeTAD/Au.

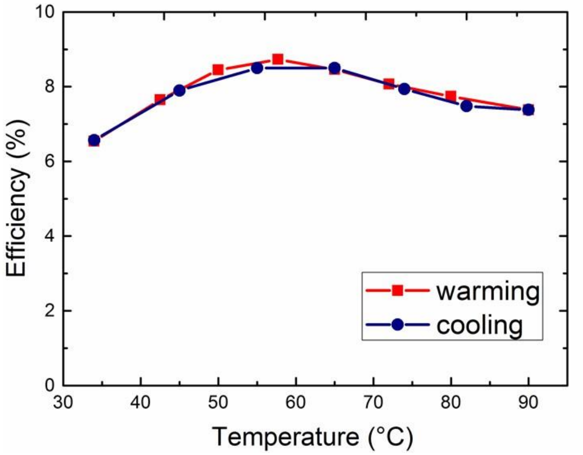

Supplementary Figure 17. Solar cell performance of a module measured under a cycling of temperature up to 90°C under ambient conditions and AM 1.5 G solar illumination at 1000 W/m^2.

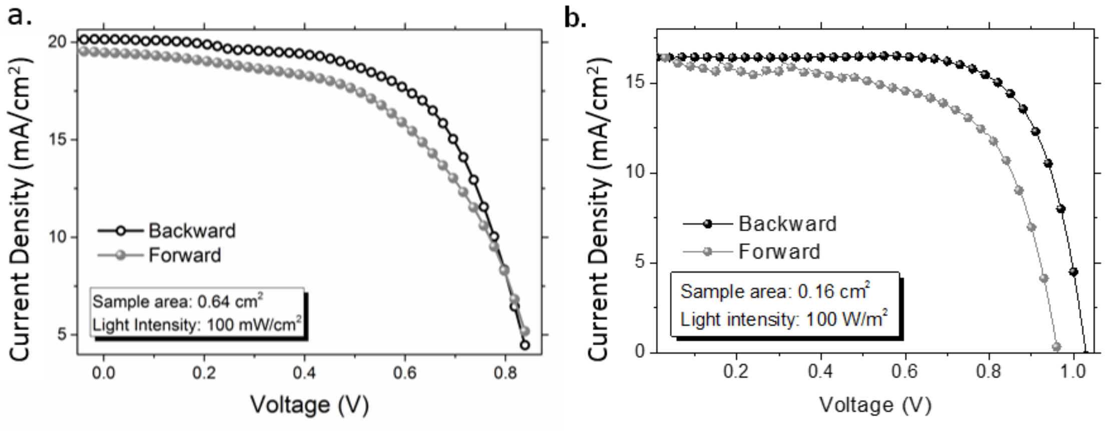

Supplementary Figure 18. a. HTM-free cell. Measurement: from 0.9V to -0.1V; divided in 50 points 200ms. 1V range /50 points; repeated 5 times. **b.** Spiro-OMeTAD cell. Measurement: from 1.1V to -0.1V; divided in 100 points 200ms. 1.2V range /100 points. Parameters are reported in Supplementary Table 4.

Supplementary Table 4. Device Parameter comparing back and forward measurement.

	J_{sc} (mA·cm^2)	***V_{oc} (V)***	***FF***	***PCE (%)***
Active area: 0.64 cm^2				
**Forward bias**	19.55	0.891	0.544	9.482
**Reverse bias**	20.15	0.883	0.608	10.804
Active area: 0.16 cm^2				
**Forward bias**	16.56	1.03	0.77	13.15
**Reverse bias**	16.56	0.98	0.62	10.06
